# Validation of the Swedish Wound‐QOL‐14: A Shortened Version of the Original 17‐Item Wound‐Specific Quality of Life Instrument

**DOI:** 10.1111/iwj.71003

**Published:** 2026-07-28

**Authors:** Ami Fagerdahl

**Affiliations:** ^1^ Department of Clinical Science and Education Södersjukhuset, Karolinska Institutet Stockholm Sweden

## Abstract

Health‐related quality of life (HRQoL) is increasingly recognized as a key outcome in the management of patients with hard‐to‐heal wounds. To assess this outcome accurately, valid wound‐specific patient‐reported outcome measures are required. The Wound‐QoL was originally developed as a 17‐item questionnaire, and a revised 14‐item version, consisting of 14 of the original 17 items, was later introduced to improve clarity and reduce respondent burden. Because changes in item composition may affect psychometric properties, the revised version requires separate evaluation. Therefore, this study aimed to assess the psychometric properties of the Swedish Wound‐QoL‐14 using data from the Swedish validation study of the Wound‐QoL‐17. This secondary analysis included data from 88 patients with hard‐to‐heal wounds recruited from an outpatient wound clinic. Responses from the original instrument were recalculated to reflect the Wound‐QoL‐14 structure. Internal consistency was assessed using Cronbach's alpha, test–retest reliability using intraclass correlation coefficients, criterion validity by comparison with EQ‐5D‐3L, responsiveness using standardized response mean, and ceiling and floor effects were examined. The Wound‐QoL‐14 demonstrated good to excellent internal consistency and test–retest reliability. Criterion validity showed moderate correlations with EQ‐5D‐3L, responsiveness was moderate for Body and Psyche and smaller for Everyday Life and the global score, and no ceiling or floor effects were observed. The Swedish Wound‐QoL‐14 is a reliable, valid, and practical instrument for assessing HRQoL in patients with hard‐to‐heal wounds in clinical practice and research.

## Key Points

- The Swedish SWAPP‐Q was successfully translated and culturally adapted for surgical patients.
- The questionnaire demonstrated good validity and high patient acceptability.
- The SWAPP‐Q can support patient participation and postoperative wound self‐management.

## 1 Introduction

Health‐related quality of life (HRQoL) has emerged as a critical parameter in both clinical practice and research concerning the treatment and management of patients with hard‐to‐heal wounds. Hard‐to‐heal wounds, such as venous leg ulcers, diabetic foot ulcers and pressure injuries, impose significant burdens on patients, not only in terms of physical discomfort but also with respect to psychological well‐being and social functioning [1, 2]. Given these multifaceted impacts, assessing HRQoL in wound care is essential for developing comprehensive treatment approaches that address not only the physiological aspects of wound healing but also the broader spectrum of patient experiences. Recent qualitative work has further emphasized the importance of patient‐centred perspectives in instrument development, highlighting how content validation ensures that questionnaires capture the lived realities of individuals with chronic wounds [3]. Moreover, guidance on how to interpret HRQoL assessments using the Wound‐QoL has been provided, underscoring its clinical relevance and utility in both practice and research [4].

To facilitate the systematic evaluation of HRQoL in individuals affected by chronic wounds, various standardized assessment instruments have been developed. Among these, the Cardiff Wound Impact Schedule (CWIS) was an early attempt to quantify the effects of chronic wounds on patients' quality of life [2]. However, recognizing the need for further refinement and improved applicability across diverse clinical settings, the Wound‐QoL was subsequently introduced as an advancement upon its predecessor [4, 5].

The original Wound‐QoL, consisting of 17 items, was rigorously validated to ensure its reliability in capturing the HRQoL aspects associated with hard‐to‐heal wounds [6]. Over the years, the instrument has undergone linguistic translation and cultural adaptation to accommodate different populations, with validation studies conducted in numerous languages such as Turkish [7], Norwegian [8] and Swedish [9]. These adaptations are crucial, as HRQoL measures must be sensitive to variations in patient demographics, healthcare settings, and sociocultural perspectives regarding chronic wound management. Importantly, qualitative validation studies such as that by Janke et al. [3] underscore the necessity of aligning questionnaire content with patient experiences, ensuring that items are not only psychometrically sound but also meaningful and acceptable to respondents.

Despite its methodological rigour, certain items within the original 17‐item Wound‐QoL have presented challenges in practical application. For example, item 12 ‘Climbing stairs has been difficult because of the wound’ has been perceived by patients as less informative, particularly in cases where they assigned a low rating to item 11, ‘I have had trouble moving about because of the wound’ [10]. Such concerns underscore the importance of patient feedback in optimizing assessment tools, ensuring that they remain relevant and reflective of actual patient experiences. In response to feedback from patients and clinicians, the original German developers undertook a revision of the instrument, culminating in a streamlined 14‐item version known as the Wound‐QoL‐14 [10]. This modified version preserves the core conceptual framework of the original instrument while addressing concerns related to item redundancy and wording that may not fully reflect the lived experiences of individuals managing hard‐to‐heal wounds. Validation studies indicate that, compared with the original 17‐item version, the Wound‐QoL‐14 demonstrates comparable or improved reliability, validity, and responsiveness, while offering greater clarity, usability, and patient acceptability [10, 11].

Despite these promising findings, modifications to an instrument's item composition may affect its psychometric properties. Therefore, evidence from validation studies of the original 17‐item version cannot automatically be generalized to the revised 14‐item version. Evaluation of the Swedish Wound‐QoL‐14 is therefore needed to ensure that the instrument maintains adequate reliability, validity, and responsiveness in Swedish patients with hard‐to‐heal wounds. The aim of this study is to assess the psychometric properties of the Swedish Wound‐QoL‐14 using data from the Swedish validation study of the original 17‐item Wound‐QoL instrument.

## 2 Methods

### 2.1 The Modified 14‐Item Wound QoL Instrument

The Wound‐QoL‐14 is a refined version of the original 17‐item Wound‐QoL, an instrument specifically designed to assess wound‐related HRQoL. The original Wound‐QoL consists of 17 items assessing impairments during the previous 7 days. Each item is scored on a 5‐point Likert scale ranging from 0 (“not at all”) to 4 (‘very much’), with higher scores indicating greater impairment and lower HRQoL. The original version categorized items into three distinct subscales: Body (items 1–5), Psyche (items 6–10), and Everyday Life (items 11–16), while item 17 was included only in the calculation of the global score. The global score is calculated as the mean of all completed items and ranges from 0 to 4.

The Wound‐QoL‐14 retains the original conceptual framework of the Wound‐QoL‐17, including the three subscales Body, Psyche, and Everyday Life, while removing three items that were identified as less informative. Specifically, item 10 (‘I have been afraid of knocking the wound’) was removed from the Psyche subscale, item 12 (‘Climbing stairs has been difficult because of the wound’) was removed from the Everyday Life subscale, and item 17 (‘The wound has been a financial burden to me’), which contributed only to the global score, was excluded from the revised instrument. Furthermore, item 5 (‘The treatment of the wound has been a burden’) was considered important for assessing HRQoL but too general to belong to the Body subscale. Therefore, it was retained only in the calculation of the global score [10].

The Wound‐QoL‐14 was developed to improve item performance, reduce respondent burden, and enhance cross‐cultural applicability while preserving the original conceptual structure of the instrument. A recent European validation study confirmed that the Wound‐QoL‐14 demonstrates good reliability, validity, responsiveness, and cross‐cultural validity in patients with hard‐to‐heal wounds. Based on these findings, the authors in that study concluded that the Wound‐QoL‐14 is preferable to the original Wound‐QoL‐17, particularly in clinical practice where time‐efficient assessment is important [12].

### 2.2 Material and Data Collection

The Swedish validation study of the original 17‐item Wound‐QoL instrument included adult patients recruited from an outpatient wound clinic in Sweden. Participants were receiving treatment for hard‐to‐heal wounds, were fluent in Swedish, and were considered cognitively able to understand and complete the questionnaire, based on an assessment by the treating healthcare professionals and the recruiting researcher [9]. Each participant completed the Swedish version of the 17‐item Wound‐QoL instrument alongside the generic HRQoL instrument, EQ‐5D‐3L, at baseline and again after a six‐week follow‐up. Data collection for the original Swedish validation study was conducted between August 2015 and July 2016.

The study was approved by the local Ethics Committee (2015/714‐31) and conducted in accordance with good clinical practice guidelines and the ethical principles outlined in the Helsinki Declaration.

### 2.3 Psychometric Analysis and Statistics

Data obtained from the completed questionnaires in the validation study of the original 17‐item Wound‐QoL instrument were systematically processed to construct a new dataset. This process involved the exclusion of three items (#10, #12, and #17) and the reorganization of domain classifications to correspond with the modified 14‐item Wound‐QoL instrument [10]. The newly adapted Swedish version, Wound‐QoL‐14, was subsequently assessed for its psychometric properties.

Reliability was evaluated using internal consistency measures and intraclass correlation (ICC) to determine test–retest stability. These assessments were based on data from patients whose HRQoL ratings remained unchanged after 6 weeks, as indicated by a transition question added in the EQ‐5D‐3L on perceived overall health status (better, unchanged, or worse). Only patients reporting unchanged health status were included in the test–retest analysis.

Criterion validity was assessed against the EQ‐5D‐3L, a generic measure of HRQoL covering five dimensions: mobility, self‐care, usual activities, pain/discomfort, and anxiety/depression. The EQ‐5D‐3L was chosen because it is a widely used and validated generic HRQoL instrument and was also used in the original study describing the development of the Wound‐QoL instrument, enabling comparison with previous findings. Criterion validity was examined by calculating correlations between the Wound‐QoL‐14 and EQ‐5D‐3L using Spearman's rho. According to Cohen, correlation coefficients between 0.30 and 0.49 were interpreted as indicating a moderate association [13].

Responsiveness was assessed through the Standardized Response Mean (SRM). To determine clinically significant changes, data were analysed by calculating the difference between baseline and the six‐week follow‐up in a subgroup of participants who reported improved health outcomes at the six‐week assessment, as reflected in the EQ‐5D‐3L questionnaire.

Ceiling and floor effects were assessed by calculating the proportion of participants who achieved the highest possible score (ceiling effect) or the lowest possible score (floor effect) for each domain and for the global score. Floor and ceiling effects are considered indicators of limited measurement range, as they may reduce an instrument's ability to detect deterioration or improvement over time. In accordance with established recommendations, a floor or ceiling effect was considered present if more than 15% of participants obtained the minimum or maximum possible score, respectively [14].

Data analysis was performed using SPSS version 23.0, with a significance level set at *p* < 0.05.

## 3 Results

Initially, 103 patients were included in the study and completed the questionnaires at baseline. The response rate after the six‐week follow‐up was 89%, with a total of 92 patients. Four patients were excluded because they were wheelchair users and unable to complete the EQ‐5D‐3L questionnaire regarding mobility, leaving 88 questionnaires for analysis in the different domains and calculations of psychometric properties. The mean age of the included patients was 67 years (range 27–96), with 64 males (73%) and 24 females (27%). All included patients had hard‐to‐heal wounds with a duration of longer than 6 weeks (Table 1).

**TABLE 1:** Participant characteristics and wound‐related data.

	*n* = 88
Gender (%)	
Male	64 (73)
Female	24 (27)
Age mean (range)	67 (27–96)
Wound duration months Mean/median (range)	10/5 (2–56)
Wound aetiology (%)	
Leg ulcer (arterial/venous)	17 (19)
Infectious wound (i.e., erysipelas)	3 (3)
Pressure injury	3 (3)
Postoperative complication	19 (22)
Traumatic wound	10 (11)
Diabetic foot ulcer	36 (42)

### 3.1 Reliability

The analysis with Cronbach's alpha coefficient showed an acceptable internal consistency, according to Streiner and Norman [15] for the domains Body (0.78), Psyche (0.89) and Everyday life (0.89). Internal consistency for the overall global score of the Wound QoL instrument had an alpha of 0.92.

The test–retest analysis was conducted on patients who reported no change in their perceived health status at the six‐week follow‐up (*n* = 31). All domains demonstrated excellent stability, with ICC exceeding 0.70: Body (0.73, 95% CI 0.42–0.88), Psyche (0.83, 95% CI 0.65–0.92), and Everyday Life (0.82, 95% CI 0.61–0.91). The overall global score of the instrument also exhibited strong reliability, with an ICC of 0.86 (95% CI 0.70–0.93).

### 3.2 Validity

Criterion validity was calculated with correlation between the overall global score and the different domains of the Wound QoL instrument with the EQ‐5D‐3L index value (TTO) and the EQ‐5D‐3L VAS scale. The correlations were moderately strong across all domains, except for the Body domain, which exhibited a slightly weaker correlation. All correlations were statistically significant. When compared with the corresponding reference scores (i.e., the correlations reported in the Swedish validation study of the original Wound‐QoL‐17) [9], the observed correlations were considered equivalent (Table 2).

**TABLE 2:** Criterion validity of the Swedish Wound‐QoL‐14 and comparison with reference scores from the Swedish validation study of the original Wound‐QoL‐17* [9].

	Wound‐QoL‐14 body	Wound‐QoL‐14 psyche	Wound‐QoL‐14 everyday life	Wound‐QoL‐14 global score
EQ‐5D‐3L index (TTO)				
rho (ref.score*)	−0.0.37 (−0.34)	−0.37 (−0.32)	−0.46 (−0.44)	−0.44 (−0.44)
*p*	< 0.01	< 0.01	< 0.01	0.04
*n*	77	81	80	81
EQ‐5D‐3L VAS				
rho (ref.score*)	−0.24 (−0.30)	−0.33 (−0.32)	−0.37 (−0.36)	−0.30 (−0.32)
*p*	0.05	< 0.01	< 0.01	< 0.01
*n*	69	74	74	75

*Note:* The asterisk (*) refers to the reference scores reported in the Swedish validation study of the original Wound‐QoL‐17. The same study population was used for both the Wound‐QoL‐17 and the subsequently derived Wound‐QoL‐14, making the comparison directly applicable.

### 3.3 Responsiveness

Internal responsiveness was assessed using SRM in the group rating improved health with the EQ‐5D‐3L after 6 weeks. The results presented in Table 3 show moderate SRM for the domains Body (0.54) and Psyche (0.51). It showed small SRM for Everyday life (0.31) and for the Global score (0.45). All results were a significant value for the change.

**TABLE 3:** Internal responsiveness for the Wound‐QoL‐14 for group rating improved health with EQ‐5D‐3L after 6 weeks.

	Baseline mean; SD	6 weeks follow‐up mean; SD	Observed change mean; SD (95% confidence interval)	*p*	SRM
Body (*n* = 25)	0.83; 0.78	0.49; 0.65	0.34; 0.63 (0.08; 0.60)	0.006	0.54
Psyche (*n* = 27)	1.53; 1.05	0.98; 0.88	0.54; 1.06 (0.12; 0.96)	0.007	0.51
Everyday life (*n* = 27)	1.39; 1.08	0.99; 1.04	0.40; 1.27 (0.10; 0.90)	0.054	0.31
Global score (*n* = 27)	1.26; 0.89	0.84; 0.78	0.43; 0.96 (0.05; 0.80)	0.015	0.45

### 3.4 Ceiling and Floor Effect

The Wound QoL instrument showed no signs of unacceptable ceiling or floor effect. No participant received neither the lowest nor the highest possible score in the overall global score (Table 4).

**TABLE 4:** Ceiling and floor effects at baseline.

	Body (*n* = 80)	Psyche (*n* = 85)	Daily life (*n* = 85)	Global score (*n* = 86)
Mean; median (range)	1.04; 0.75 (0–3.50)	1.72;1.507 (0–4)	1.43; 1.17 (0–4)	1.40; 1.13 (0.06–3.82)
Floor effect % (*n*)	14 (11)	4 (3)	9 (8)	0 (0)
Ceiling effect % (*n*)	0 (0)	5 (6)	2 (2)	0 (0)

## 4 Discussion

This study evaluated the psychometric properties of the Swedish Wound‐QoL‐14 using data from the original Swedish validation cohort of the 17‐item version [9]. The results demonstrate that the Swedish Wound‐QoL‐14 maintains strong reliability, acceptable validity and adequate responsiveness, comparable to the original 17‐item instrument and consistent with findings from the revision study [10].

Importantly, the present findings are also highly consistent with those reported by Hitchman et al. [11], who evaluated the validity, reliability, and responsiveness of the Wound‐QoL‐14 in patients with diabetes‐related foot ulcers. In both studies, the instrument demonstrated good to excellent internal consistency, strong test–retest reliability, and acceptable criterion validity when compared with the EQ‐5D‐3L. The Cronbach's alpha values and ICCs observed in the current study closely mirror those reported by Hitchman et al. [11] reinforcing the robustness and stability of the Wound‐QoL‐14 across different wound populations and clinical settings.

The psychometric performance of the Swedish Wound‐QoL‐14 aligns closely with results reported by von Stülpnagel et al. [10] and the original Swedish validation of the Wound‐QoL‐17 [9]. Cronbach's alpha values and ICCs observed in this study fall well within the range reported in international versions, further supporting the cross‐cultural applicability and reliability of the instrument.

The slightly weaker correlation observed for the *Body* domain in relation to EQ‐5D‐3L is consistent with the findings by Hitchman et al. [11] and may reflect inherent conceptual differences between generic and disease‐specific HRQoL instruments. While EQ‐5D‐3L captures broad aspects of health status, the Wound‐QoL‐14 focuses specifically on wound‐related symptoms and burdens, which may not be fully reflected by a generic measure. This interpretation aligns with previous research emphasizing the complementary roles of generic and wound‐specific HRQoL instruments.

The removal of items 10, 12 and 17 appears to have enhanced the practical applicability of the instrument without negatively affecting its psychometric properties. Notably, Hitchman et al. [11] also reported high patient acceptability of the Wound‐QoL‐14, describing the questionnaire as easy to understand and not overly burdensome. The exclusion of item 12, which was previously perceived by some patients as insensitive or irrelevant in certain mobility contexts, may have contributed to improved acceptability and relevance in both studies.

The reassignment of item 5 to the global score only represents a more conceptually appropriate approach, as treatment burden is a multidimensional construct not confined to physical symptoms [16]. This modification supports a more patient‐centred and theoretically grounded measurement strategy, consistent with contemporary HRQoL methodology.

Responsiveness analyses demonstrated moderate sensitivity to change in the *Body* and *Psyche* domains and smaller responsiveness for *Everyday Life* and the global score, a pattern also observed by Hitchman et al. [11]. These results likely reflect the relatively short follow‐up period of 6 weeks, which may be insufficient to capture meaningful changes in daily functioning and social participation in patients with hard‐to‐heal wounds. Nonetheless, the instrument was able to detect statistically significant changes among patients reporting improved health status, supporting its clinical utility for monitoring outcomes over time.

The absence of ceiling and floor effects further supports the suitability of the Wound‐QoL‐14 for use across a broad spectrum of wound severity, mirroring findings from Hitchman et al. [11] and other international validation studies [7, 8].

Overall, the Swedish Wound‐QoL‐14 appears well‐suited for implementation in routine wound care due to its brevity, clarity, and patient‐friendly design. Reduced respondent burden may contribute to higher completion rates and improved feasibility in both clinical practice and research settings.

From a research perspective, the instrument provides a reliable and valid outcome measure for evaluating wound therapies, care models, and rehabilitation strategies. Its demonstrated comparability with both the Wound‐QoL‐17 [5, 9] and the findings reported by Hitchman et al. [11] allows continuity with existing literature while offering a more efficient and practical assessment tool.

### 4.1 Strengths and Limitations

A major strength of this study is the use of an established validation dataset with standardized data collection and longitudinal follow‐up. The inclusion of both reliability and responsiveness analyses provides a comprehensive evaluation of the instrument's performance. However, limitations include the single‐centre design and relatively small sample size, which may affect generalizability. Additionally, the six‐week follow‐up period may have constrained the ability to fully assess long‐term responsiveness.

Future studies should explore the Wound‐QoL‐14 in larger, more diverse populations and across different care settings, including home care and inpatient services.

The Swedish version of the Wound‐QoL‐14 demonstrates strong psychometric properties and represents a reliable, valid, and practical tool for assessing HRQoL in patients with hard‐to‐heal wounds. The refined item structure improves usability while preserving measurement accuracy, supporting its integration into both clinical practice and research. Further studies are encouraged to confirm its performance in broader populations and longer follow‐up periods.

## Funding

The author has nothing to report.

## Ethics Statement

An ethical approval was obtained by the local Ethics Committee (2015/714‐31). The original Wound‐QoL‐17 study was conducted in accordance with the principles of the Declaration of Helsinki, ensuring respect for participants, protection from harm, and fairness. In the original study, participants received written information about the study's purpose, procedures, anonymity, voluntariness, and their right to withdraw, and provided written informed consent prior to participation. The present study is a secondary analysis based solely on recalculated data derived from the original dataset, with no additional data collection involving participants.

## Conflicts of Interest

The author declares no conflicts of interest.

## Data Availability

The data that support the findings of this study are available from the corresponding author upon reasonable request.
