# Correction to ‘Validation of the Swedish Wound‐QOL‐14: A Shortened Version of the Original 17‐Item Wound‐Specific Quality of Life Instrument’

**DOI:** 10.1111/iwj.71017

**Published:** 2026-08-09

A. Fagerdahl , “Validation of the Swedish Wound‐QOL‐14: A Shortened Version of the Original 17‐Item Wound‐Specific Quality of Life Instrument,” International Wound Journal 23, no. 8 (2026): e71003, 10.1111/iwj.71003.42521241 PMC13413224

The Key Points section in the published article was incorrect. The originally published Key Points did not accurately reflect the study findings. The Key Points should be corrected to read as follows:

- The Swedish Wound‐QoL‐14 showed good reliability and validity.
- The instrument effectively captures wound‐specific health‐related quality of life.
- Wound‐QoL‐14 is a practical tool for clinical practice and research.

We apologize for this error.

